# Comparison of Pinoresinol and its Diglucoside on their ADME Properties and Vasorelaxant Effects on Phenylephrine-Induced Model

**DOI:** 10.3389/fphar.2021.695530

**Published:** 2021-08-09

**Authors:** Yiqiong Pu, Yiqing Cai, Qi Zhang, Tianling Hou, Teng Zhang, Tong Zhang, Bing Wang

**Affiliations:** ^1^ Experiment Center of Teaching and Learning, Shanghai University of Traditional Chinese Medicine, Shanghai, China; ^2^ School of Pharmacy, Shanghai University of Traditional Chinese Medicine, Shanghai, China; ^3^ Yueyang Hospital of Integrated Traditional Chinese and Western Medicine, Shanghai University of Traditional Chinese Medicine, Shanghai, China; ^4^ Clinical Research Institute of Integrated Medicine, Shanghai Academy of Traditional Chinese Medicine, Shanghai, China; ^5^ Department of Pharmaceutical Sciences, School of Pharmacy, University of Pittsburgh, Pittsburgh, PA, United States; ^6^ Center for Pharmaceutics Research, Shanghai Institute of Materia Medica, Chinese Academy of Sciences, Shanghai, China

**Keywords:** pinoresinol, pinoresinol diglucoside, ADME, pharmacokinetic, vasorelaxation

## Abstract

Pinoresinol (PINL) and pinoresinol diglucoside (PDG), two natural lignans found in *Eucommia ulmoides* Oliv. (Duzhong), have several pharmacological activities. However, there is no report available on their absorption, distribution, metabolism, and elimination (ADME) properties. Given the possible wide spectrum of their application in therapeutic areas, this area should be investigated. This work studied the *in vitro* ADME properties of PDG and PINL, including their kinetic solubility, permeability across monolayer cells (PAMPA), protein binding, and metabolic stabilities in liver microsomes. The *in vivo* pharmacokinetic study and *in vitro* vasorelaxant effects on isolated phenylephrine-induced aortic rings of PINL and PDG were also investigated. It was found that both of their kinetic solubility in PBS (pH 7.4) was greater than 100 μM, indicating that they are both soluble compounds. The permeability investigations (*P*_*eff*_) by PAMPA indicated that PINL had higher permeability than PDG (*p* < 0.05). Both components represented moderate plasma protein binding activities (average binding rate in human plasma: PINL 89.03%, PDG 45.21%) and low metabolic rate (*t*_*1/2*_ in human liver microsome: PINL 1509.5 min, PDG 1004.8 min). Furthermore, the results of pharmacokinetic studies indicated that PINL might be eliminated less quickly than PDG from the rat plasma, and its cumulative urinary excretion was much lower than that of PDG. The phenylephrine-induced aortic rings demonstrated concentration-dependent vasorelaxation in PDG, PINL, or their combination group. The vasorelaxant effects of PINL were more obvious than those of PDG, whereas the vasorelaxant effect of the combinations was significantly better than that of the single component (*p* < 0.05). The similarity or difference between PINL and its diglucoside in these pharmaceutical aspects may offer valuable insights into the further exploration of lignans and might contribute to relevant studies involving natural products with similar molecular structure and their glucosides.

## Introduction

Cortex Eucommiae (called *Duzhong* in Chinese), a kind of Chinese medicinal herb, is the dried barks of *Eucommia ulmoides* Oliv., which is often used as an anti-hypertension agent in clinical practice (Greenway et al., 2011). A Chinese patent drug named QuanDuzhong Capsules (containing Duzhong extracts only), has been included in the current *Chinese Pharmacopeia* (2020 Edition), where anti-hypertension is listed as its main indications. Additionally, Cortex Eucommiae has been found effective in mild osteoarthritis (Ahn et al., 2019), has anti-hyperuricemia (Fang et al., 2019) and neuroprotection properties (Kwon et al., 2011), and has also been used in the management of bone loss (Qi et al., 2019), rheumatoid arthritis (Xing et al., 2020), and cognitive deficits (Kwon et al., 2013).

In the study on the components contained in the herb, the natural products identified from Cortex Eucommiae include lignans, iridoids, phenolics, flavonoids, steroid, and terpenoids among which lignans and their derivatives are the main components. In Cortex Eucommiae, the lignans mainly include bisepoxylignans, monoepoxylignans, neolignans, and sesquilignans (He et al., 2014). Bisepoxylignans are recombined compositions whose side chains of two phenylpropanoids between 7–9 and 7′–9′ are connected to form a tetrahydrofuran ring. The tetrahydrofuran ring is combined with a *cis* stereo configuration, and the optical rotation is mostly dextral (Wang et al., 2019).

**FIGURE 1:**
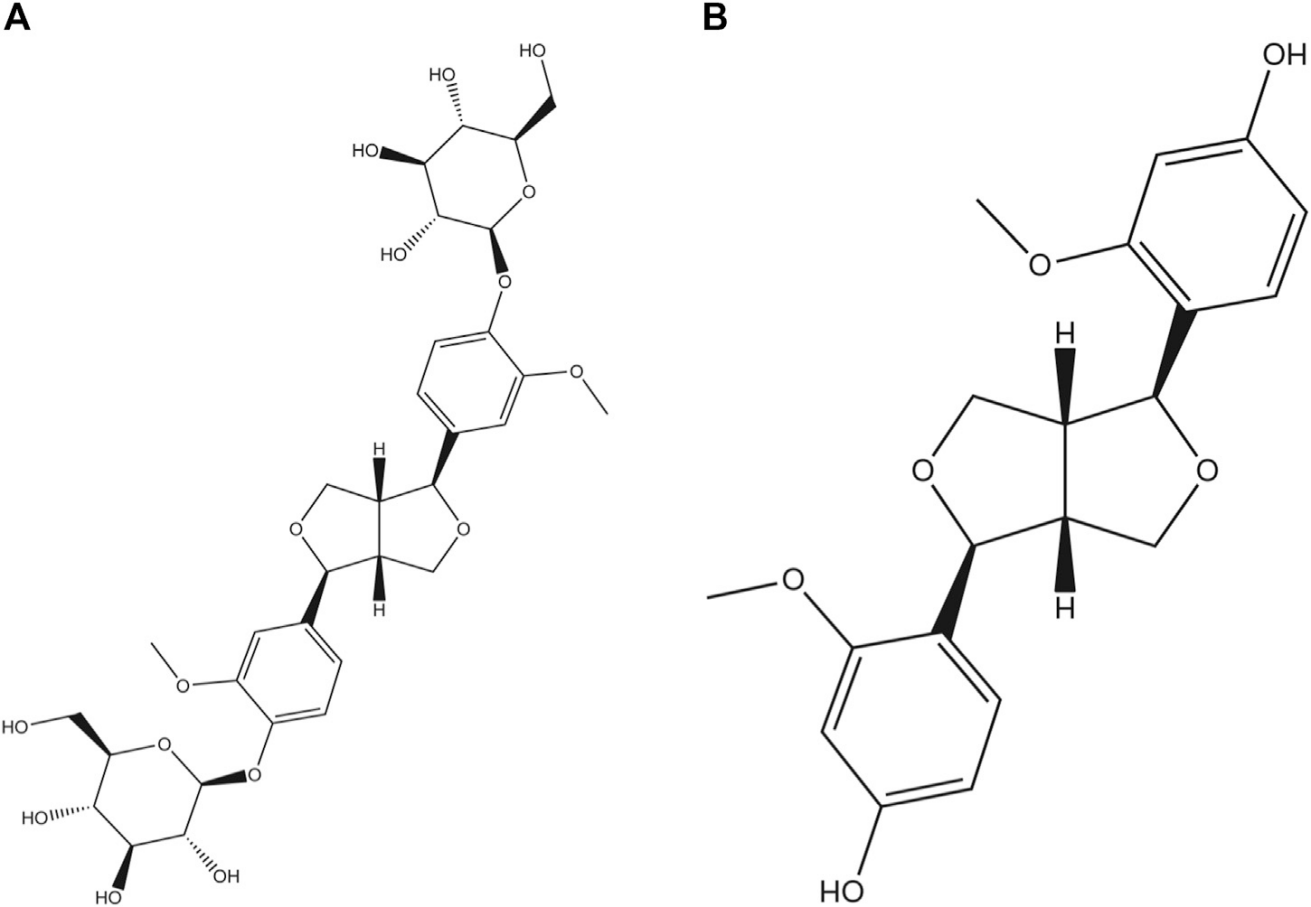
Molecular structures. **(A)** PDG and **(B)** PINL.

Among the found natural bisepoxylignans, pinoresinol diglucoside (PDG, Figure 1A), (+)-1-pinoresinol 4,4′-di-O-β-D-glucopyranoside, was used as a quality control marker for Cortex Eucommiae by high performance liquid chromatography (HPLC) in Chinese Pharmacopoeia (2020 edition). PDG can attenuate cardiac hypertrophy *via* AKT/mTOR/NF-κB signaling in pressure overload-induced rats (Chen et al., 2021) and can attenuate neuroinflammation, apoptosis, and oxidative stress in a mice model with Alzheimer’s disease (Lei et al., 2021). Pinoresinol (PINL, Figure 1B) is another natural lignan found in Cortex Eucommiae. According to recent reports, PINL can ameliorate Aβ-induced memory deficits without sensory dysfunction (Yu et al., 2021), promote osteoblastic proliferation (Jiang X. et al., 2019), protect rat pial microcirculation from hypoperfusion and reperfusion (Lapi et al., 2015), and ameliorate CCl_4_-induced acute liver injury (Kim et al., 2010).

Given that both PDG and PINL are plant lignans (or derivatives) found in Cortex Eucommiae, they are first found and produced *via* the phenylpropanoid pathway in plants (Zhang et al., 2015a). Some reports also support the bioconversion between them (Zhang et al., 2015b; Zhu et al., 2018). Moreover, from the perspective of the abovementioned pharmacological effects, PINL and its diglucoside (PDG) are likely to share some similar bioactions, such as effects on the nervous system. Therefore, their responses on the same model, such as vasorelaxant effects on phenylephrine-induced model, are worth investigating.

Although there are proven pharmacological effects of PDG and PINL, their absorption, distribution, metabolism, and elimination (ADME) properties have not been fully reported yet. Given the wide spectrum of their further application in those therapeutic areas, it is vital to investigate their ADME properties, especially in the early stages of drug development (Kour et al., 2016). In absorption studies, the focus is usually on intestinal permeability as the major determinant of absorption, where a common approach is to measure the *in vitro* permeation properties. Additionally, it is generally assumed that only free drug can cross membranes and bind to the intended molecular target, and binding of drug molecules to plasma proteins can affect drug metabolism, excretion, disposition, and efficacy (Trainor, 2007). Therefore, only unbound drugs can be excreted by the kidney *via* urine; thus, the unbound drug to plasma proteins is a key parameter in predicting volume of distribution, metabolism and clearance, developing pharmacokinetics/pharmacodynamics relationships, and predicting clinical dosages (Lombardo et al., 2002). Meanwhile, human serum albumin (HSA) is the most abundant plasma protein and the major drug binding protein, and thus, drug-HSA interaction mechanisms have been widely investigated by molecular docking studies (Zhang et al., 2016; Liu et al., 2020). Metabolic stability is one of the most important ADME properties of drug candidates that may affect clearance, half-life, and oral bioavailability.

Therefore, in this paper, *in vitro* ADME properties of the lignans, including their kinetic solubility in PBS, permeability ability across the monolayer cells (PAMPA), protein binding, and metabolic stabilities in liver microsomes were investigated using molecular modeling approach. Subsequently, an *in vivo* pharmacokinetic study was performed on male Sprague-Dawley (SD) rats via oral and intravenous administrations. Furthermore, the *in vitro* vasorelaxant effects on isolated phenylephrine-induced aortic rings of PINL and PDG were investigated because of the well-documented antihypertensive activity of Cortex Eucommiae. The outcome of the study may provide evaluable information about the early ADME properties, pharmacokinetic performance of PINL and its diglucoside (PDG), and their *in vitro* vasorelaxant effects related with anti-hypertension. The similarity or difference found between PINL and its diglucoside in these pharmaceutical aspects may offer valuable information for the further exploration of the lignans and might also contribute to the relevant study involving the natural products with similar molecular structure and their glucosides.

## Materials and Methods

### Drugs

Pinoresinol diglucoside (PDG) (purity >98%, 111537–200502) were obtained from National Institutes for Food and Drug Control (Beijing, China). Pinoresinol (PINL) (purity>98%, No. BBP00347) were obtained from Yunnan Biobiopha Biological Technology Co. (Yunnan, China). Tolbutamide, propranolol, omeprazole and sodium dichlorophenolate were purchased from Sigma-Aldrich Co. (St. Louis, Mo, United States). Midazolam was purchased from Shanghai Tuolei Chemical Reagent Co. (Shanghai, China). Ketoconazole and cyclosporin-A were obtained from Shanghai Gaolan Laboratory Co. (Shanghai, China). Nicardipine was obtained from National Institutes for Food and Drug Control (Beijing, China). Dextromethorphan and fluorescite were purchased from Adamas-beta Reagents Co., Ltd. (Shanghai, China). Amprenavir was purchased from Shanghai Bide Pharmatech Co., Ltd. (Shanghai, China). Phenylephrine (PE) was purchased from Tokyo Chemical Industry Co., Ltd. (Tokyo, Japan). Acetylcholine chloride (Ach) was purchased from Sigma-Aldrich Co., Ltd. (St. Louis, MO, United States).

### Reagents

Rat liver microsomes and human liver microsomes were purchased from Sekisui XenTech Co. (Kansas City, KS, United States). Human plasma was obtained from Rild Research Institute for Liver Disease Co. (Shanghai, China). Glucose-6-phosphate dehydrogenase (G-6-PD) and NADP were purchased from Sigma-Aldrich Co. (St. Louis, Mo, United States). Dulbecco’s modified eagle’s medium (DMEM), trypsogen and fetal bovine serum (FBS) were purchased from Invitrogen Co. (Grand Island, NY, United States). D-glucose-6-phosphate sodium salt (G-6-P) was purchased from Aladdin Industrial Co. (Shanghai, China). Dimethyl sulfoxide (DMSO) and methanol (chromatographic grade) were purchased from Sigma-Aldrich Co. (St. Louis, Mo, United States). Acetonitrile (chromatographic grade) was purchased from Thermo Fisher Scientific Co. (Waltham, MA, United States). The other chemicals used in this study were of analytical grade, and purchased from Sinopharm Chemical Reagent Co. (Shanghai, China).

### Animals

Male Sprague-Dawley (SD) rats (180–260 g) used in pharmacokinetic study were provided by Shanghai Super-BK Laboratory Animal Co. Ltd. (Shanghai, China), with Production License Number of SCXK (Shanghai) 2013–0016, and Laboratory Animal Certificate Number of 2008001656325, 2008001656835, 2008001657135. All animal experiments were carried out according to the Ethics Committee Guidelines for Experimental Animal Care of Shanghai University of Traditional Chinese Medicine (Shanghai, China).

SD rats (180–260 g), three male and three female, were used in the vasorelaxant effects investigation, provide by Shanghai SLAC Laboratory Animal Co., Ltd. (Shanghai, China). The protocol of was approved by the Institutional Animal Care and Use Committee at Yueyang Hospital, Shanghai University of Traditional Chinese Medicine (YYLAC-2019–018–3).

### Kinetic Solubility Test in PBS

Kinetic solubility assay is the method of choice for determining the maximum solubility of a compound under most reactions or assay conditions (Ekins et al., 2015). The kinetic solubility in PBS was determined via precipitation methods with slight modifications (Ekins et al., 2015; Kosugi and Hosea, 2021). The test samples (PDG and PINL) and reference (nicardipine and ketoconazole) were dissolved in dimethyl sulfoxide (DMSO) and diluted by phosphate-buffer saline (PBS, pH 7.4) to obtain the final concentration of 100 μg/ml. Internal standard (IS) solutions (propranolol and tolbutamide) were dissolved by acetonitrile (50 ng/ml propranolol + 200 ng/ml tolbutamide).

The sample solutions were analyzed using LC-MS/MS. All the samples (test samples and references) for detection were prepared in duplicate. The conditions for LC-MS/MS are shown in Support Materials (Supplementary Table S1).

The Solvent Accessibility method in Discovery Studio (Accelrys, San Diego, CA, United States) was employed to calculate the SASA of PDG and PINL with 960 grid point per atom (other parameters were set as default).

### Parallel Artificial Membrane Permeability Assay

PAMPA was performed in a 96-well PAMPA pre-coated filter plate (#353015, Corning Gentest) as described previously (Balimane et al., 2005; Cui et al., 2015). Verapamil and digoxin solutions, used as reference, prepared in the same manner as the test drugs. Tolbutamide dissolved with acetonitrile (200 ng/ml) was used as IS.

The resultant supernatants were determined by LC-MS/MS. The specific assay conditions are shown in Support Materials (Supplementary Table S2).

The effective permeability coefficient (*P*_*e*_) was calculated as follows:

$$Pe=-2.303\frac{V_{a}\;V_{d}}{\left(V_{a}+V_{d}\right)A_{t}}lg\left(1-\frac{\left(V_{a}+V_{d}\right)C_{a}\left(t\right)}{V_{d}\;C_{a}\left(0\right)\left(1-R\right)}\;\right)$$ (1)

$$R=1-\frac{C_{d}\left(t\right)\times V_{d}+C_{a}\left(t\right)\times V_{a}}{C_{0}\times V_{d}}=1-\frac{C_{d}\left(t\right)\times 0.3+C_{a}\left(t\right)\times 0.2}{C_{0}\times 0.3}$$ (2)

where *V*_*a*_ is the volume of the acceptor compartment (0.2 ml), *V*_*d*_ is the donor volume (0.3 ml), *C*_*a*_ *(0) and C*_*d*_ *(0)* are the initial concentrations (mg/ml) of the acceptor and donor compartment, respectively, *C*_*a*_ *(t)* and *C*_*d*_ *(t)* are the acceptor and donor concentrations (mg/ml) of the samples at the incubation time, respectively, *A* is the artificial lipid membrane area (0.3 cm^2^), and *t* is the incubation time (18,000 s).

### Plasma Protein Binding Rate

The plasma protein binding rate of the samples was investigated using rapid equilibrium dialysis method (Waters et al., 2008; Jiang C. et al., 2019; Yuan et al., 2020). In brief, the unbound drugs (or references) were separated from plasma (human and rat) in a 96-well plate with a semipermeable membrane through which only unbound drug can permeate. The equilibrium unit was sealed and incubated at 37°C in a mixer at 100 rpm for 4 h.

The drugs (PDG and PINL) and reference (diclofenac and propranolol) stock solutions (10 mM) were prepared by dissolving them in DMSO and further diluting with acetonitrile-water (70:30, *v/v*), yielding their respective working solution (0.1 mM). Then, 50 ng/ml of midazolam and 50 ng/ml of tolbutamide (in acetonitrile) were used as IS.

After reaching equilibrium, the concentrations of the samples in both the plasma and the buffer compartment were determined. According to the specification, the resultant supernatant was collected, properly diluted by distilled water, vortexed for 10 min, and finally analyzed using LC-MS/MS. All the samples (PDG, PINL, diclofenac, and propranolol) for detection were prepared in duplicate. The LC-MS/MS conditions for determining the concentrations of PDG and PINL are shown in Support Materials (Supplementary Table S3).

The concentrations of the samples were determined by internal standard methods, in which the data were acquired and analyzed by Analyst 1.6.1 software station (AB Sciex Corporation, Boston, MA, United States). The equation of plasma protein binding rate is calculated as follows:

$$Free\;drug\;rate\;\left(\%\right)=\frac{C_{B}}{C_{A}}\times 100\%$$ (3)

Plasma protein binding rate (%)=100%−Free drug rate(%)  (4)

*C*_*B*_ is the drug concentration in dialysis chamber, and *C*_*A*_ is the drug concentration in the sample chamber.

The protein structure of HSA was extracted from the crystal structure of HSA obtained from the Protein Data Bank in Europe (PDB ID: 1ao6) with water molecule deleted. The molecular structures of PDG and PINL were obtained from the PubChem of National Center for Biotechnology Information (https://pubchem.ncbi.nlm.nih.gov) (PubChem CID: 174003, 73399). The molecular dynamics program GROMACS 4.0.5 was employed for energy minimization calculations. The docking program AutoDock Vina 1.1.2 was used to perform the automated molecular docking calculation (Trott and Olson, 2010). Energy minimization protocol was first executed to prepare the PDG and PINL molecular models. After the heavy atom of HSA was fixed, the flexible PDG and PINL molecules were docked. The docking results with the relatively low energy were used for successive structure optimization. In the minimization protocol, all the models were placed in vacuum. The non-bond cutoff distance of 18.5 Å, spline width of 1.0 Å, and buffer width of 0.5 Å were used. In the docking protocol, a Lamarckian Genetic Algorithm (LGA) in combination with a grid-based energy evaluation method was used for pre-calculating grid maps according to the interatomic potentials of all atom types present in the host and guest molecules, including the Lennard-Jones potentials for van der Waals interactions and Coulomb potentials for electrostatic interactions. A grid map of dimensions 70 Å × 70 Å × 70 Å, with a grid spacing of 0.375 Å, was placed to cover the classical active site of HSA. AutoDockTools (Version 1.4.5, The Scripps Research Institute, La Jolla, CA, United States) was used to calculate the atomic partial charges via the Gasteiger–Marsili method (Gasteiger and Marsili, 1980). The parameters used for the global search were an initial population of 50 individuals, with a maximal number of energy evaluations of 1,500,000 and a maximal number of generations of 50,000 as an end criterion. Other docking parameters were set as default.

### Metabolic Stability in Liver Microsomes

Metabolic stability investigation of liver microsomes was performed as previously reported (Jenkins et al., 2004). First, 2 μL of working solution (0.25 mM omeprazole, 0.25 mM dextromethorphan, 1.25 mM PDG, and 1.25 mM PINL) was added in a 96-well plate containing 398 μL of RLM and HLM diluents. Then, this incubation plate containing mixed solution and NADPH regeneration system was agitated on a plate shaker at 100 rpm and pre-incubated at 37°C for 5 min. Subsequently, 300 μL of precooled quenching solution was added to each well of another deep-well plate (quench plate), which was cooled on ice. The solution in the incubation plate was transferred into the well of the quench plate as the samples in 0 min before it was mixed with another 20 µL of NADPH regeneration solution in the wells. The final incubation solutions contained 5 µM test drugs (PDG, PINL) or 1 µM reference compounds (omeprazole and dextromethorphan) and 0.5 mg/ml of microsomes (total protein).

The incubation plate was incubated at 37°C for 0, 10, 30, and 90 min. At each time point, 100 μL of the supernatant in the incubation plate was removed from each well, quenched immediately, transferred to the wells of the ice-cold quench plate, and vortexed for 5 min. Additionally, an equal volume of pre-warmed fresh regeneration solution was added to the incubation plate for compensation after every withdrawal. Finally, after centrifugation at 5,000 × g for 15 min at 4°C, the supernatants of the quenched plate were diluted by distilled water and analyzed by an LC-MS/MS system. Each test was operated in duplicate. The determination conditions of LC-MS/MS are shown in Support Materials (Supplementary Table S4).

The *in vitro* T_1/2_ was determined by converting the analyte/IS peak area ratios to percentage of drug remaining using the initial peak (t = 0 min) area ratio values as 100%. The equations of *T*_*1/2*_ and protein concentration are shown as follows:

$$T_{1/2}=-\frac{0.693}{k}$$ (5)

$$CL_{int}=\frac{0.693}{T_{1/2}}\times \frac{V}{M}$$ (6)

where *k* is the slope of the linear regression from *log* percentage remaining versus incubation time relationships, and *V/M* is the 1/(protein concentration).

### Pharmacokinetics and Excretion Studies

PDG and PINL were dissolved in a solution consisting DMSO, Solutol HS 15, and normal saline (1:3:16, *v/v/v*) at a concentration of 1 mg/ml (used in intravenous administration groups) and 4 mg/ml (used in oral administration groups).

Eighteen male SD rats were housed in the barrier rodent facilities to acclimatize for a week in animal quarters, which had air conditioning and an automatically controlled photoperiod of 12 h of light daily. Rats had free access to standard chow diet and water until 12 h prior to the beginning of the experiment, at which time only water was accessed freely. The rats were randomly divided into six groups, with three rats in each group. Groups 1 and 2 received PDG and PINL intravenously at a dose of 5 mg/kg by tail vein bolus injection (IV, with dose volume of 5 ml/kg) for a single-dose administration. Groups 3 and 4 were given PDG and PINL at 40 mg/kg by oral gavage administration (PO, with dose volume of 5 ml/kg) for a single dose. The rats in these groups (groups 1–4) were used to determine their plasma samples for the pharmacokinetic study of the tested drugs (PDG and PINL). Groups 5 and 6 were used to determine their urine samples for the excretion study *via* with tail vein bolus injection (IV, with dose volume of 5 ml/kg) of PDG and PINL.

The stock standard solutions (1.0 mg/ml) of tested drugs (PDG and PINL) and IS (diclofenac sodium for PINL urine samples and tolbutamide for other samples) were prepared by dissolving the components in DMSO and vortexing. A series of calibration standard working solutions (20.0, 10.0, 2.0, 1.0, 0.2, 0.04, and 0.02 μg/ml) was prepared freshly by diluting the stock solution stepwise using ACN-water (70:30, *v/v*). The quality control (QC) working solutions of PDG and PINL were diluted to the concentration of 16.0, 0.8, and 0.06 μg/ml. PINL was determined in urine samples by preparing the calibration standard working solutions (20.0, 10.0, 2.0, 1.0, 0.4, 0.2, and 0.1 μg/ml) in the same manner. The IS working solutions of 200 ng/ml of tolbutamide or 50 ng/ml of diclofenac sodium were prepared by dilution of the stock standard solution with acetonitrile solution. All the solutions were stored at 4°C and brought to room temperature before use.

For plasma samples, the calibration standards were prepared by spiking 3 μL of the appropriate standard working solutions of PDG (or PINL) to 57 μL of blank rat plasma to prepare the final plasma concentration of PDG (or PINL) ranging from 1 ng/ml to 2000 ng/ml. QC samples (3, 40, 800, and 1,600 ng/ml) were prepared separately. For PDG urine samples, the calibration standards were prepared by spiking 5 μL of the appropriate standard working solutions of PDG to 95 μL blank rat urine to prepare the final urine concentration of PDG ranging from 1 ng/ml to 2000 ng/ml. QC samples (3, 40, 800, and 1,600 ng/ml) were prepared separately. For PINL urine samples, the calibration standards were prepared by spiking 5 μL of the appropriate standard working solutions of PINL to 95 μL blank rat urine to make final urine concentration of PINL ranging from 5 ng/ml to 2000 ng/ml. QC samples (15, 30, 400, and 1,600 ng/ml) were prepared separately.

Blood samples were collected via orbital venous plexus puncture into heparinized microfuge tubes at intervals of 0, 0.083, 0.25, 0.5, 1, 2, 4, 8, 10, 24, and 30 h after administration. The plasma samples were obtained by centrifuging the blood samples at 6,000 rpm for 10 min at 4°C and storing them at −20°C until further analysis. For the plasma calibration standards, QC plasma samples, and the testing plasma samples, 40 µL of the plasma samples were extracted with 120 µL of IS working solution (tolbutamide) by vortex mixing for 1 min and at 4°C centrifugation for 10 min at 13,000 rpm. The supernatant was separated and evaporated under a gentle stream of nitrogen. The residue was reconstituted with 120 µL of 30% ACN followed by vortex mixing. Then, the solutions were centrifuged at 13,000 rpm for 10 min at 4°C. The supernatant was injected into autosampler vials, 10 µL (PDG) or 20 µL (PINL), for LC-MS/MS.

Urine samples were collected while the rats remained isolated in metabolic cages at intervals of 0, 2, 4, 8, 24, and 30 h after administration, and the volumes of the urine were measured. The urine samples were also stored at −20°C before tested by LC-MS/MS. For the urine calibration standards, QC urine samples, and the testing urine samples, 100 µL of the urine samples were extracted with 300 µL of methyl tert-butyl ether (MTBE) by vortex mixing for 1 min and centrifuged at 4°C for 10 min at 13,000 rpm. Then, 200 µL of the supernatant was separated and evaporated under a gentle stream of nitrogen. The residue was reconstituted with 120 µL of 30% ACN with 5 µL of IS working solution (5 ng/ml diclofenac sodium), followed by vortex mixing for 10 min. Then, the samples were injected into autosampler vials, 10 µL (PDG) or 5 µL (PINL), for LC-MS/MS.

The chromatographic separations of the analytes were achieved in a Thermo Betasil C18 analytical column (2.1 mm × 50 mm, 5 µm) with an Agilent 1,200 system and the mass detector of AB Sciex Qtrap 5,500 system. Linear gradient elution was performed with certain portions of 10 mM ammonium acetate-water solution containing 0.1% formic acid (mobile phase A) and methanol (mobile phase B). The detailed conditions have been described in Support Materials (Supplementary Table S5).

### Vasorelaxant Effects on Isolated PE-Constricted Aortic Rings

SD rats were anesthetized by exposure to ether, and the thoracic aorta was removed and immersed in Krebs-Henseleit (K-H) buffer (Lee et al., 2013) at 4°C. The connective tissues and fats around the aorta were carefully removed under the microscopes, and the blood was flushed. The aorta ring was cut into four sections approximately 3 mm long and suspended in organ chambers containing K-H buffer. The endothelium was also removed by gently rubbing the lumen of the vessel with a cotton swab. The aortic rings were placed in a 5 ml organ chambers of DMT multiwire myograph system (620M, Danish Myo Technology, Denmark) and were filled with K-H buffer aerated with 95% O_2_ and 5% CO_2_ at 37°C. The aortic rings were mounted on hooks connected to a tension transducer and a displacement device.

The initial vessel tension was slowly adjusted to 15 mN and maintained balanced for 60 min. The buffer was renewed once every 15 min. After equilibration, the vessel viability was pre-constricted by exposing the aortic rings to 60 mM KCl for 10 min (Ding et al., 2019). Then, the K-H buffer was continuously renewed to elute and restore the initial tension of the vascular ring to assay the vascular activity. The pre-treated operation was repeated two times, where the constriction amplitude after each KCl stimulation to reach the previous constriction amplitude was taken as the criterion, before the experiment continued. Subsequently, 10^–6^ M PE was added to contract the blood vessel. After the constriction was stable, different concentrations of Ach (10^−9^–10^–5^ M) were used to dilate the vessels. Then, the vasorelaxation degree could be calculated with different Ach concentrations, as the following equation:

$$Vasorelaxation\;degree\;\left(\%\right)=\frac{Maxium\;constriction\;tension\;induced\;by\;PE-Minium\;diastolic\;tension\;induced\;by\;Ach}{Maxium\;constriction\;tension\;induced\;by\;PE-Resting\;tension}\times 100\%$$ (10)

When the vasorelaxation degree corresponding to Ach (10^–5^ M) is greater than 80%, the vascular endothelium is considered intact and functional. The endothelium is considered to be removed with a vasorelaxation degree of less than 20% (Lee et al., 2013). Endothelium-intact and endothelium-denuded aortic rings from the same aorta were tested.

After equilibration, the endothelium-intact and the endothelium-denuded aortic rings were pre-constricted with 10^–6^ M PE in the K-H buffer system. When the constriction was maintained, the PDG and PINL solutions were added sequentially to obtain the different final concentrations (25, 50, 75, 100, 125, 150, 175, and 200 μM), and the corresponding vasoconstrictive responses were examined and recorded.

After equilibration, the endothelium-intact aortic rings were pre-constricted with 10^–6^ M PE in the K-H buffer system. After the stable constriction, PDG, PINL, and their mixture (1:1, *m/m*) solutions were added sequentially to obtain the different final concentrations (12.5, 25, 37.5, 50, 62.5, 75, 82.5, and 100 μM). Then, the corresponding vasoconstrictive responses were examined and recorded.

### Statistical Analysis

In the ADME properties and pharmacokinetics studies, data are presented as mean ± standard deviation (SD). Statistically significant differences were analyzed by one-way ANOVA or Student’s *t*-test. The level of significance was set at *p* < 0.05. The LC/MS/MS data were collected and analyzed by Analyst 1.6.1 (AB Sciex). Pharmacokinetic analyses were performed by non-compartmental methods using Phoenix WinNolin version 6.3 (Pharsight Corporation, Mountain View, CA, United States).

In the vasorelaxant effects investigations, all the data are presented as mean ± SEM. Statistical analysis was performed using two-way ANOVA followed by Fisher’s least significant difference (LSD) test. Values at *p* < 0.05 were considered statistically significant.

## Results

### Kinetic Solubility in PBS

**FIGURE 2:**
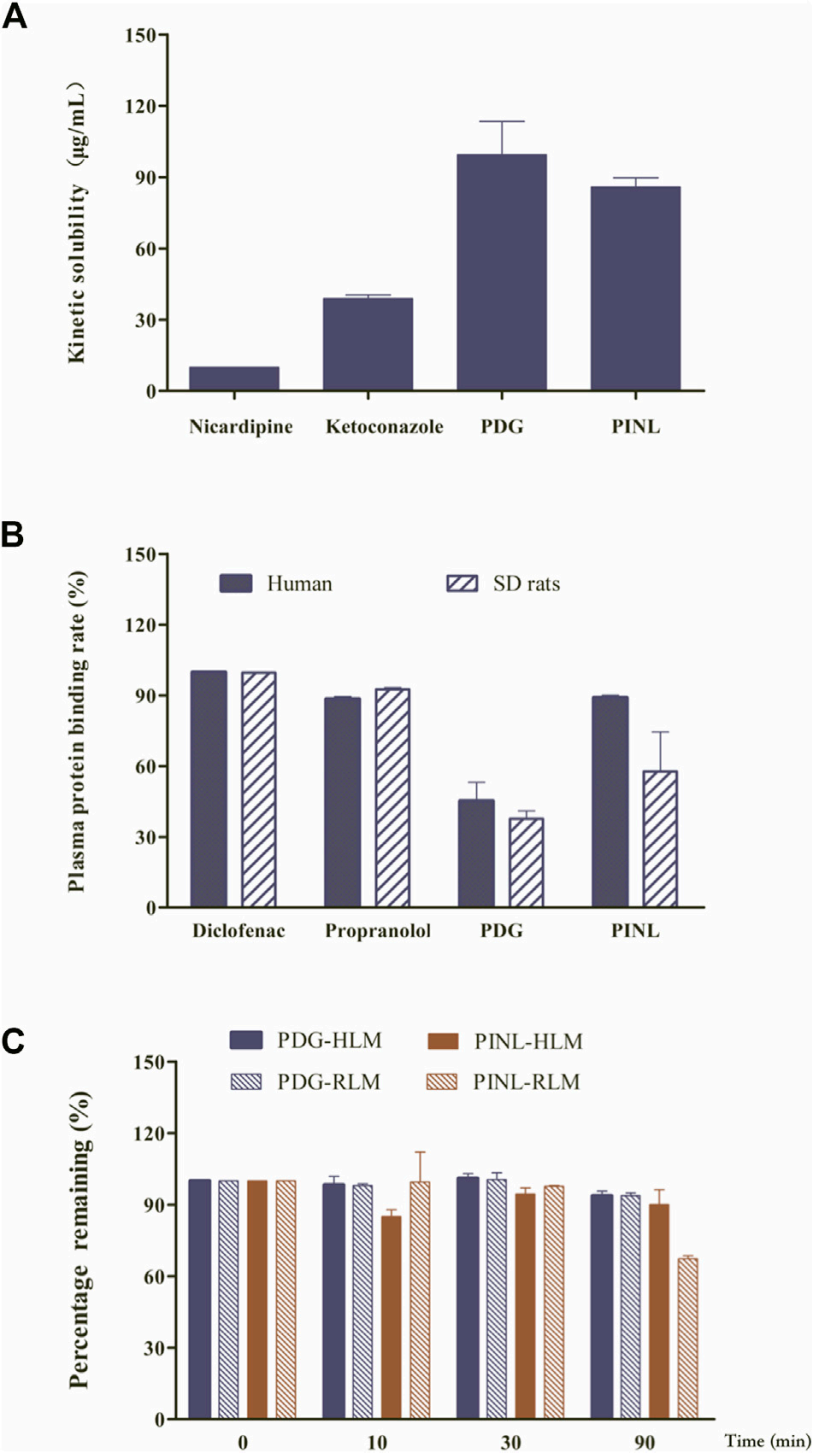
**(A)** Results of kinetic solubility test of the compounds in PBS (pH 7.4). **(B)** Results of plasma protein binding. **(C)** Results of metabolic stability investigation in human liver microsomes (HLM) and rat liver microsomes (RLM) (*n* = 2).

The results of kinetic solubility assay are shown in Figure 2A. The solubility of the reference compounds (nicardipine and ketoconazole) in PBS (100 mM, pH 7.4) were 9.94 ± 0.05 and 38.91 ± 1.54 μg/ml, respectively, which were similar to previously reported results (Kerns and Di, 2005). The kinetic solubilities of PDG and PINL were 99.49 ± 14.01 μg/ml (approximately 146 µM) and 85.87 ± 3.84 μg/ml (approximately 240 µM), respectively.

**FIGURE 3:**
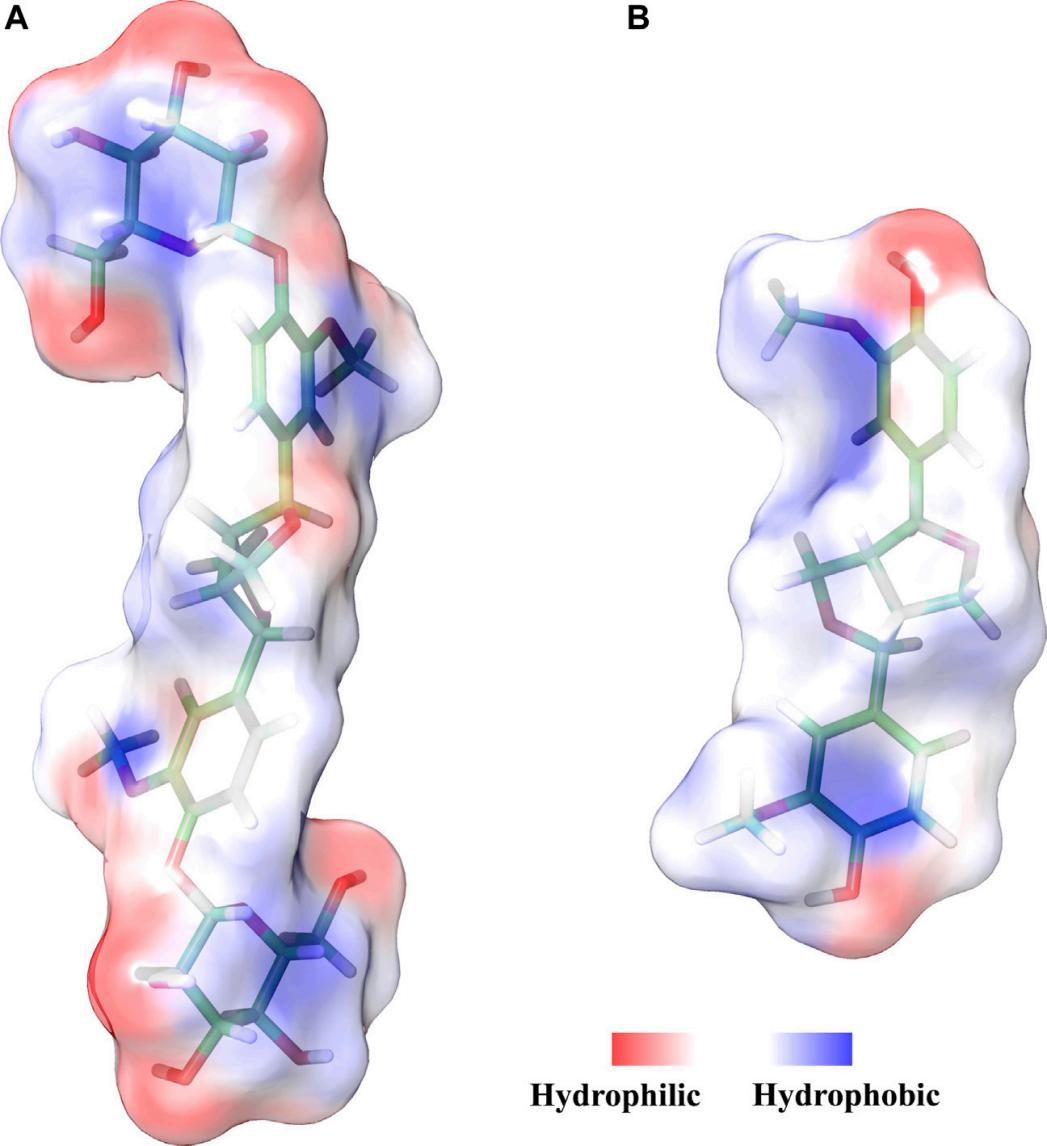
Hydrophilic/hydrophobic schematic of **(A)** PDG and **(B)** PINL with surface.

The modeling results (Figure 3) showed that the calculated solvent accessible surface areas (SASA) of PDG and PINL were 938 Å^2^ and 565 Å^2^, respectively. In terms of molecular structure, PDG can be considered PINL plus two β-D-glucopyranose at both ends, which increases the total SASA by 373 Å^2^. Therefore, in polar solvents, such as PBS, the solubility of PDG is obviously higher than PINL in experiments, which is consistent with the experimental results (an increase of approximately 13.62 μg/ml).

## Parallel Artificial Membrane Permeability Assay

In this study, digoxin and verapamil with low and high permeability, respectively, were used as reference. The permeability of PDG and PINL was assessed and compared with the reference. The results of the PAMPA are shown in Table 1. *P*_*eff*_ was 0.02 × 10^–7^ cm/s for PDG and 176.84 × 10^–7^ cm/s for PINL, which showed a significant difference. As such, PINL had higher permeability than PDG in the *in vitro* investigation.

**TABLE 1:** Effective permeability coefficients of the tested samples (*n = 2*).

Sample (Reference)	*P*_*eff*_ (cm/s, ×10^–7^)	Permeability CV (%)
Digoxin	5.89	40.6
Verapamil	271.36	5.4
PDG	0.02	23.8
PINL	176.84	7.7

### Plasma Protein Binding Rate

In this study, the protein binding rates of the references (diclofenac and propranolol) are shown in Figure 2B and were in accordance with the reported rates (Waters et al., 2008). This result confirmed that the assay system is reliable for the determination of protein binding for the compounds. The results of plasma protein binding rate of PINL and PDG in the concentrations of 10 µM are shown in Figure 2B, which shows that the average binding rates of PINL and PDG in human plasma were 89.03 and 45.21%, and their average binding rates in rat plasma were 57.74 and 37.69%, respectively. There was an apparent difference between the plasma protein binding rates of PINL in human and rat plasma. However, the plasma protein bindings were relatively similar in human and rat plasma, both of which were of moderate binding.

**FIGURE 4:**
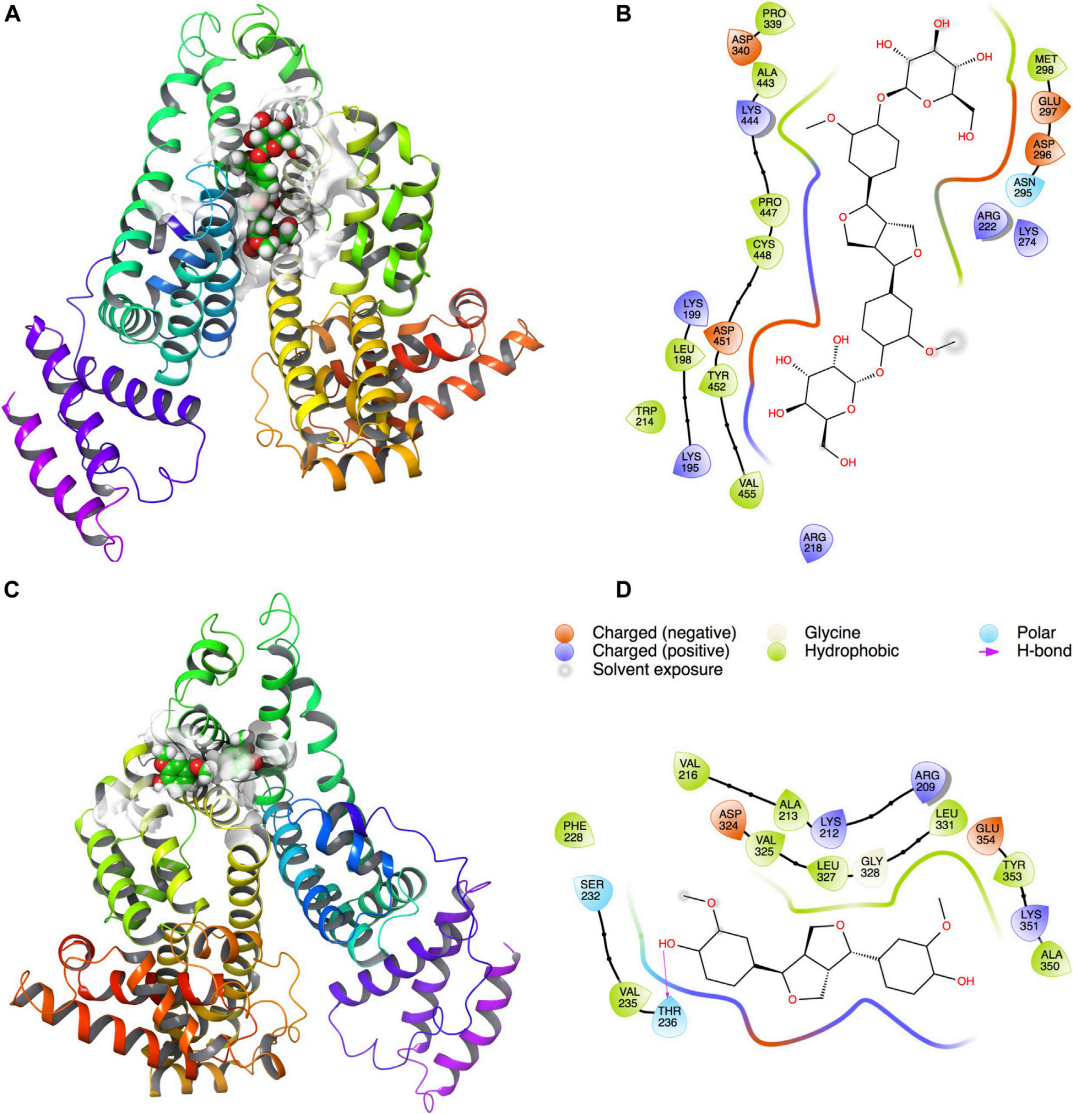
PDG and PINL on the possible site of HSA. **(A)** PDG on HSA; **(B)** Ligand interaction diagram of PDG with HSA; **(C)** PINL on HSA; **(D)** Ligand interaction diagram of PINL with HSA.

Docking results showed that PINL and PDG had been docked to the classical hydrophobic portion of the HSA molecule (Figure 4). The docking free energy was −8.4 and −7.9 kcal mol^−1^, respectively. One of the hydrophilic–OH groups in PINL formed a hydrogen bond with the residue of THR236 in HSA. This interaction may cause the docking free energy increase of approximately 0.5 kcal mol^−1^ and was consistent with the trend of binding rate data between PINL and HSA in real experiments.

### Metabolic Stability in Liver Microsomes

The metabolic stability of liver microsomes of the references (dextromethorphan, omeprazole) and compounds (PDG, PINL) are shown in Table 2. The remaining amounts (%) to specific incubation time are shown in Figure 2C. Dextromethorphan and omeprazole showed supposed metabolism performance in HLM and RLM as reported (Obach, 1999; Walsky and Obach, 2004), which indicated that the assay system could be used to verify the *in vitro* metabolic stability of PDG and PINL. In HLM and RLM with NADPH, the *t*_*1/2*_ of PINL were respectively 1,509.5 min (25.2 h) and 149.7 min (2.5 h) at the microsomal incubation concentration of 0.5 mg/ml of microsomal proteins, with *Cl*_*int*_ of 0.9 μL/min/mg and 9.3 μL/min/mg. In addition, in HLM and RLM with NADPH, the *t*_*1/2*_ of PDG were respectively 1,004.8 min (16.7 h) and 1,035.2 min (17.3 h), with *Cl*_*int*_ of 1.4 and 1.3 μL/min/mg. In the incubation time of 90 min, all the remaining amounts of PDG in HLM and RLM exceeded 90%. The remaining amount of PINL in HLM was also high (90.1 ± 6.2%). These results indicated the low metabolic rate of PDG and PINL, either in HLM or RLM.

**TABLE 2:** Results of metabolic stability of HLM and RLM (*n* = 2).

Reference/Compound	HLM		RLM	
	T_1/2_ (min)	CL_int_ (µL/min/mg)	T_1/2_ (min)	CL_int_ (µL/min/mg)
Dextromethorphan	63.8	21.7	—	—
Omeprazole	—	—	15.6	88.7
PINL	1,509.5	0.9	149.7	9.3
PDG	1,004.8	1.4	1,035.2	1.3

### Pharmacokinetic and Excretion Studies

**FIGURE 5:**
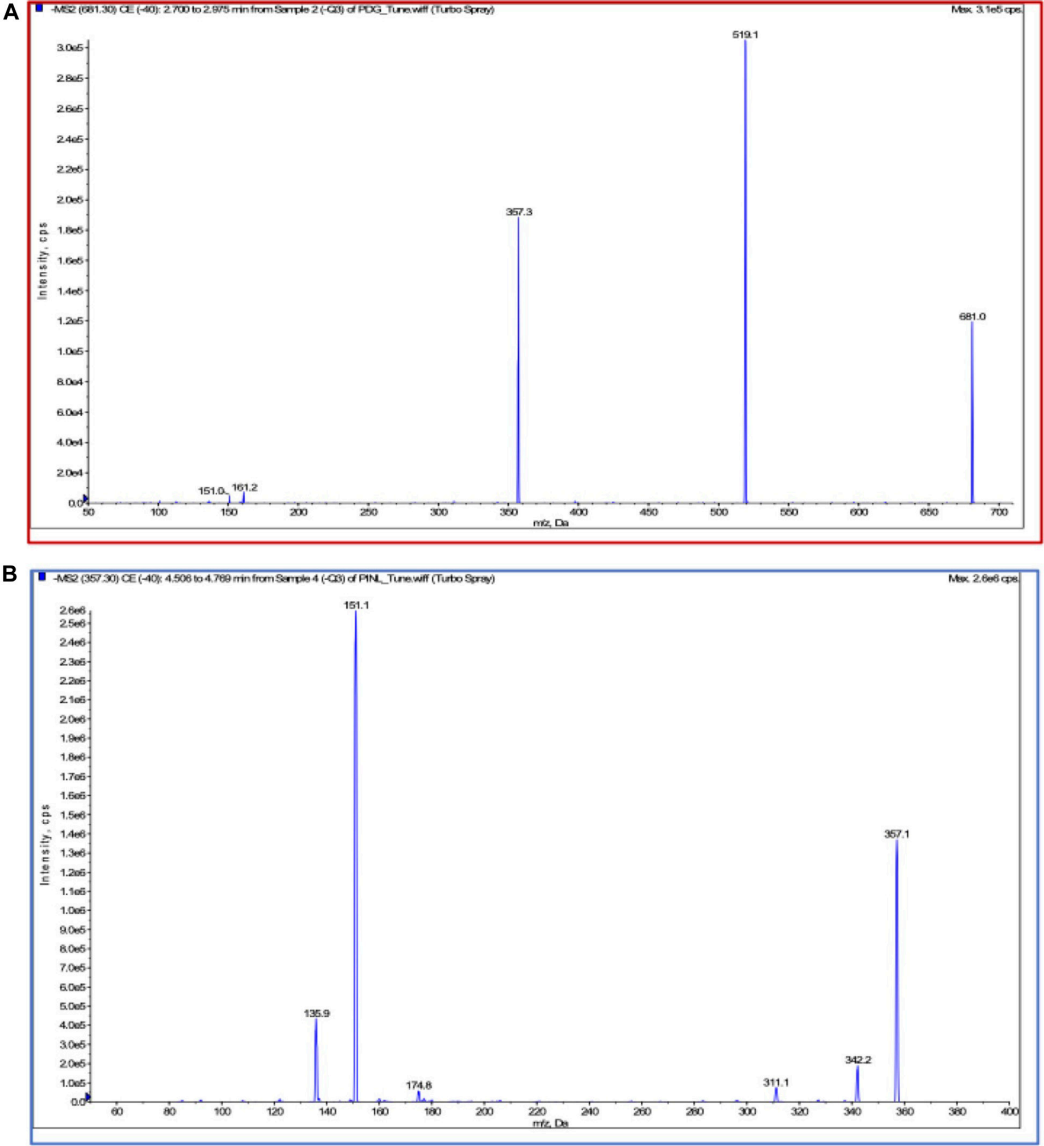
Product spectra and fragmentation reaction in ESI (-) mode. **(A)** PDG; **(B)** PINL.

**FIGURE 6:**
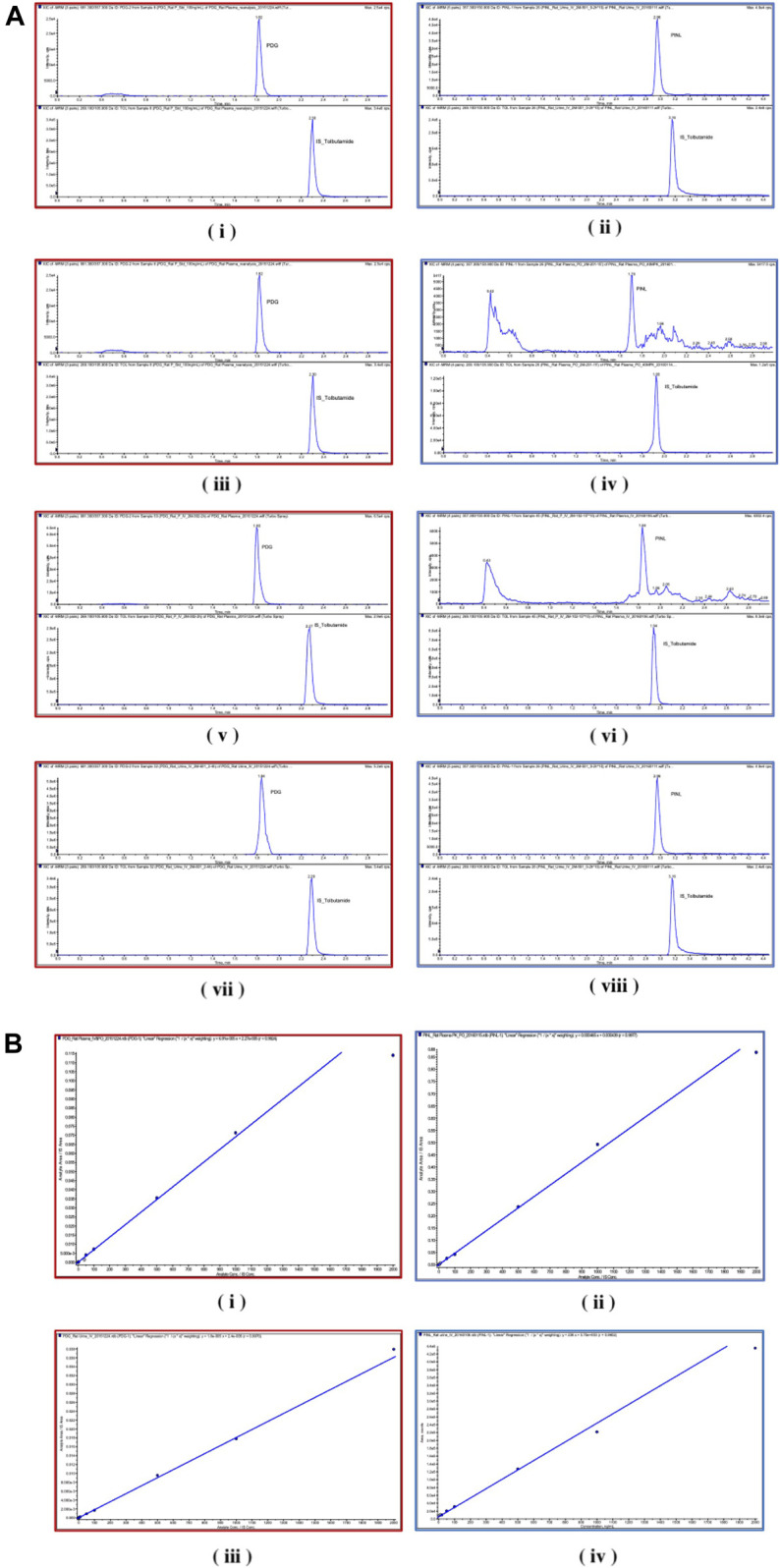
**(A)** Chromatograms. **(i)** PDG and **(ii)** PINL in blank rat plasma samples; **(iii)** PDG and **(iv)** PINL in rat plasma after a single dose of intravenous administration (5 mg/kg); **(v)** PDG and **(vi)** PINL in rat plasma after a single dose of oral administration (40 mg/kg); **(vii)** PDG and **(viii)** PINL in rat urine after a single dose of intravenous administration (5 mg/kg). **(B)** Standard curves. **(i)** PDG and **(ii)** PINL in rat plasma; **(iii)** PDG and **(iv)** PINL in rat urine.

**FIGURE 7:**
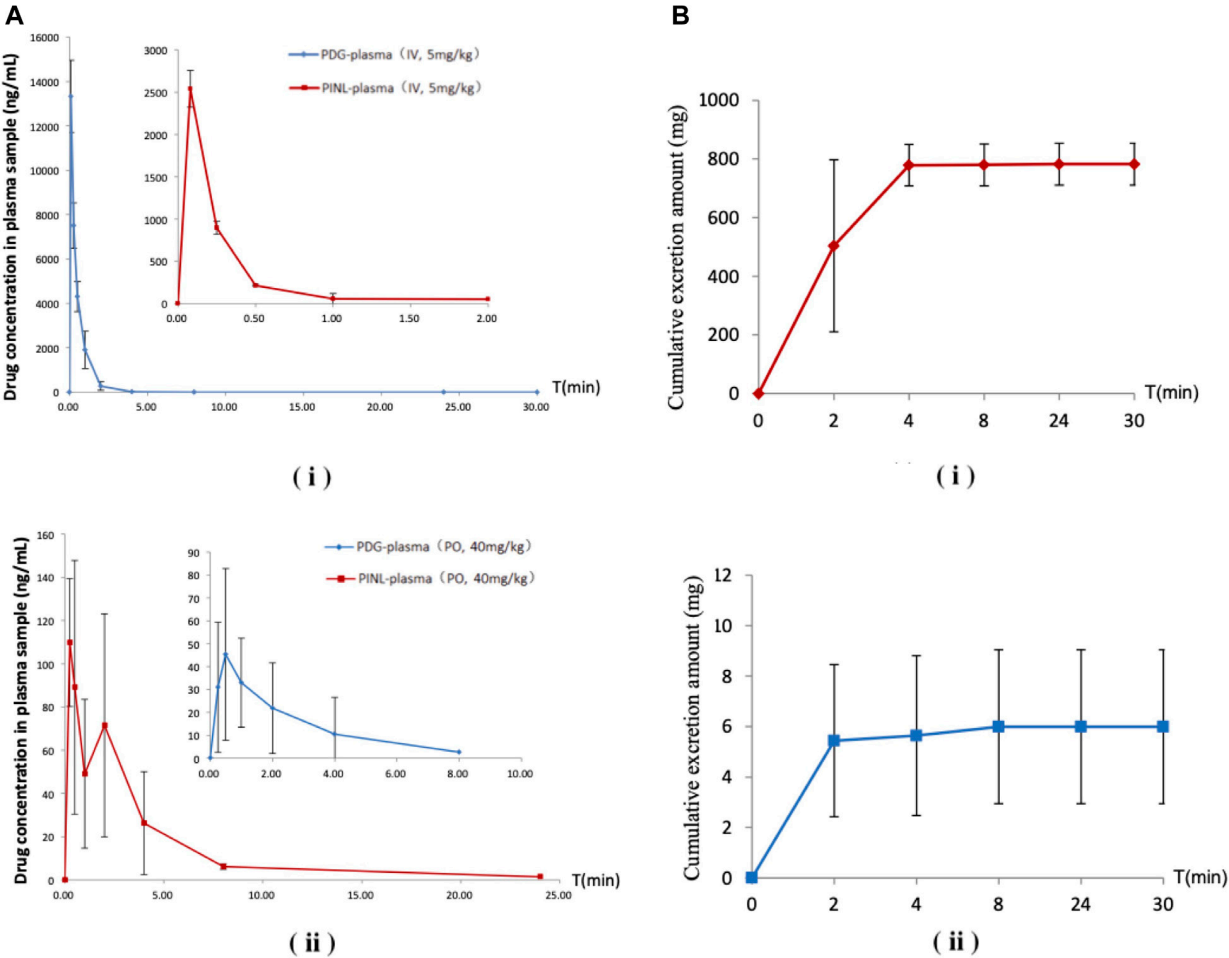
**(A)** Mean plasma concentration–time profiles after a single-dose administration. **(i)** Intravenous administration; **(ii)** Oral administration (*n = 3*). **(B)** Cumulative excretion in urine samples after intravenous administration. **(i)** PDG; **(ii)** PINL.

Chromatographic and mass spectrometric conditions were optimized to enhance the signal response of analytes and improve peak efficiency. The validated method was successfully applied to the pharmacokinetic study of PDG and PINL after intravenous and oral administration at a single dose, in which the MRM mass spectrum of PDG and PINL in negative ESI mode (Figure 5), typical chromatograms of the compounds in rat plasma or urine samples (Figure 6A), and their standard curves (Figure 6B) were investigated. The linearity was obtained by plotting the peak area ratios of the compound (PDG or PINL) to IS against the corresponding concentrations and estimated by weighted least-squares linear regression using *1/x* ^*2*^ as weighting factor. The acceptance criterion of a calibration curve was a correlation coefficient (*r*) of 0.99 or better. The correlation coefficients (*r* > 0.99) of the calibration curves showed good linearity over the concentration ranges as shown in Table 3. The mean plasma concentration–time profiles of PDG and PINL after a single dose of intravenous or oral administration are shown in Figure 7A. Based on the plasma concentrations, the corresponding pharmacokinetic parameters calculated using non-compartmental analysis is listed as mean ± SD from individual rats shown in Table 4.

**TABLE 3:** Regression equations and linear ranges for the determination of PDG and PINL in rat plasma and urine.

Compound	Administration	Biosample	Y = aX + b	*r*	Linear range (ng/ml)
PDG	IV (5 mg/kg)	Plasma	Y = 6.91 × 10^−5^X + 2.27 × 10^–5^	0.9924	1–2,000
		Urine	Y = 1.53 × 10^−5^X + 2.76 × 10^–5^	0.9934	1–2,000
	PO (40 mg/kg)	Plasma	Y = 1.40 × 10^−3^X + 1.37 × 10^–4^	0.9989	1–2,000
PINL	IV (5 mg/kg)	Plasma	Y = 1.04 × 10^−5^X + 9.44 × 10^–5^	0.9951	1–2,000
		Urine	Y = 8.38 × 10^−4^X + 2.75 × 10^–2^	0.9939	1–2,000
	PO (40 mg/kg)	Plasma	Y = 4.65 × 10^−4^X + 4.39 × 10^–4^	0.9977	1–2,000

**TABLE 4:** Pharmacokinetic parameters of PDG and PINL in plasma samples after intravenous (mean ± SD, *n* = 3) and oral (*n* = 3) administration by non-compartmental analysis.

Parameter	PDG (IV) (5 mg/kg)	PDG (PO) (40 mg/kg)	PINL (IV) (5 mg/kg)	PINL (PO) (40 mg/kg)
HL_Lambda_z (hr)	2.0 ± 0.5	1.15 ± 0.75	0.34 ± 0.35	3.57 ± 2.87
T_max_ (h)	0.1 ± 0.0	0.50 ± 0.00	0.08 ± 0.00	0.33 ± 0.14
C_max_ (ng/ml)	13,328.57 ± 1,625.4	45.3 ± 37.5	2,538.77 ± 217.7	113.1 ± 35.3
AUC_last_ (h×ng/mL)	7,466.2 ± 1,635.7	113.0 ± 121.9	805.0 ± 35.2	317.0 ± 192.0
AUC_INF_pred_ (h × ng/mL)	7,466.5 ± 1,635.8	121.1 ± 128.9	825.0 ± 54.1	332.4 ± 192.1
MRT_INF_pred_ (h)	0.51 ± 0.09	1.90 ± 0.95	0.26 ± 0.18	3.83 ± 1.66
Vz__pred_ (L/kg)	1.98 ± 0.43	777.0 ± 392.4	2.83 ± 2.81	789.0 ± 608.7
Cl__pred_ (L/h/kg)	0.69 ± 0.15	653.9 ± 492.6	6.08 ± 0.40	151.1 ± 84.3

Following intravenous administration, the maximum measured plasma concentration (*C*_*max*_) was 13,329 ng/ml (19.56 nM) and occurred at 0.1 h, while oral administration of PDG at 40 mg/kg resulted in *C*_*max*_ of 45.3 ng/ml (0.0665 nM) at 0.5 h. As for PINL, the measured *C*_*max*_ was 2,538.8 ng/ml (7.10 nM) after intravenous administration at the first blood sampling time of 0.08 h. Meanwhile oral administration of PINL resulted in *C*_*max*_ of 113.1 ng/ml (0.317 nM) at 0.33 h.

Thereafter, the PDG concentrations in oral administration decreased with the elimination *t*_*1/2*_ values of 1.15 h, indicating that PDG could be eliminated rapidly from the rat plasma, while the PINL elimination *t*_*1/2*_ values were 3.57 h.

The absolute oral bioavailability (*F%*) of PDG was approximately 0.19%, calculated from area under curve (*AUC*_*last*_) values in concentration-time curves, from intravenous and oral administration. The absolute oral bioavailability *F%* of PINL after oral administration was 4.93%.

The cumulative excretion amounts of PDG and PINL in rat urine within 30 h after intravenous administration are shown in Table 5 and Figure 7B. The cumulative urinary excretion of PINL was much lower than that of PDG, that is only 0.60% of the dose for 30 h, while the latter was approximately 78.24% of the dose (5 mg/kg).

**TABLE 5:** Mean cumulative excretion amount of PDG and PINL in urine samples after intravenous administration (*n* = 3).

Time (h)	PDG (IV, 5 mg/kg) (ng)	PINL (IV, 5 mg/kg) (ng)
0–2	503,669.8 ± 292,869.4	5,440.0 ± 3,011.9
2–4	274,878.3 ± 236,625.8	201.7 ± 203.0
4–8	989.1 ± 1,053.6	351.4 ± 565.5
8–24	2,596.9 ± 1776.4	ND^a^
24–30	245.1 ± 173.0	ND^a^
Wash	25,410.3 ± 5,874.3	ND^a^
Cumulative Excretion Amount (ng)	807,789.5 ± 65,828.8	5,993.1 ± 3,046.5
Cumulative Excretion Rate (%)	0.062 ± 0.005	0.0005 ± 0.0003

a ND, Not detected.

### Vasorelaxant Effects on the Isolated Aortic Rings

**FIGURE 8:**
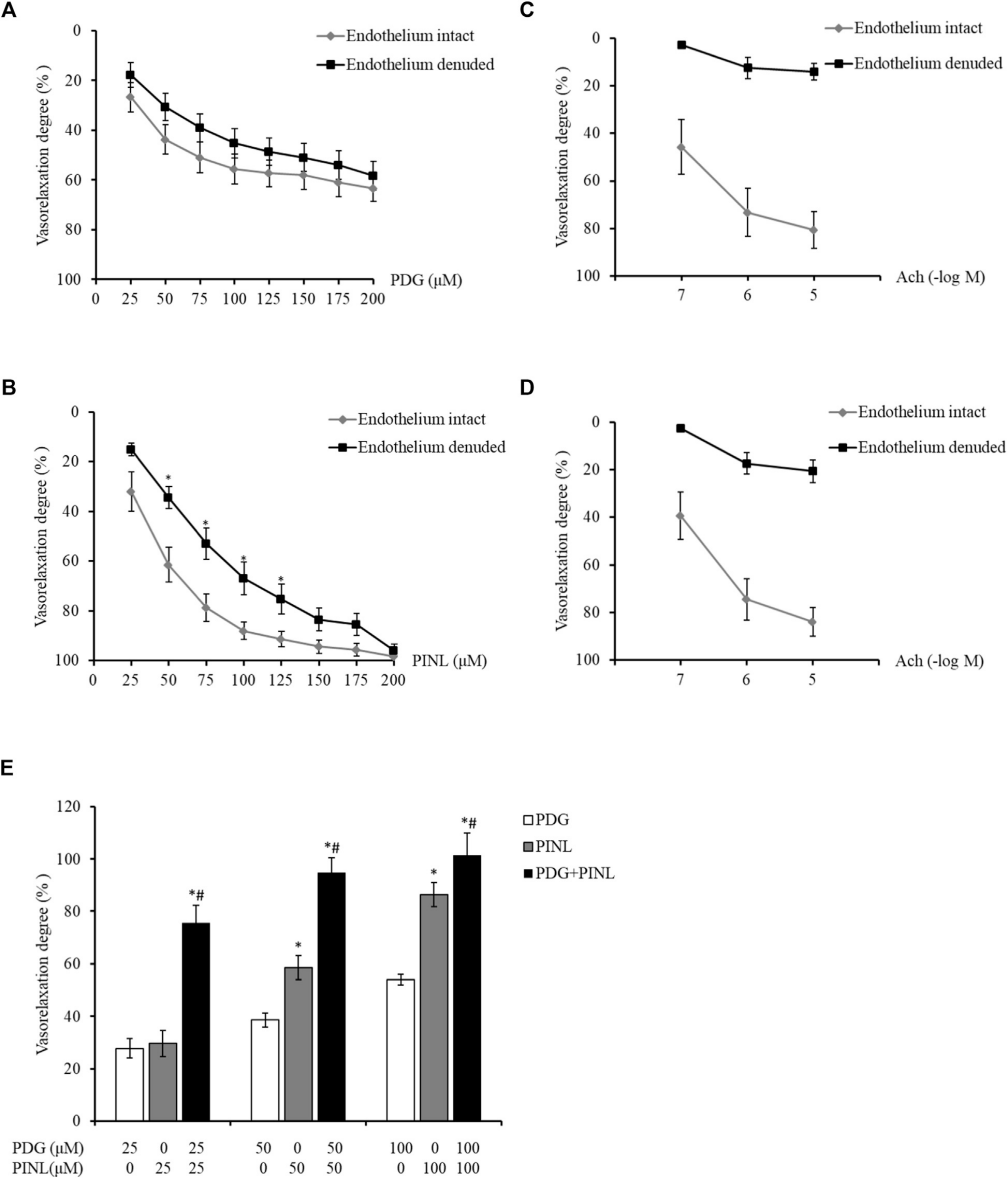
**(A)** Vasorelaxant effects on the PE-constricted aortic rings of the PDG group (*n = 6*). **(B)** Vasorelaxant effects on the PE-constricted aortic rings of the PINL group (*n = 6*). ^*^ *p* < 0.05, *vs.* endothelium intact group. **(C)** Vasorelaxant response to Ach from the aortic rings of the PDG group. **(D)** Vasorelaxant response to Ach from the aortic rings of the PINL group (*n = 6*). **(E)** Comparison of the three groups (*n = 6*). ^*^ *p* < 0.05, *vs.* PDG group, ^#^ *p* < 0.05, *vs.* PINL group.

The results showed that with the increased concentration of PDG, the aortic rings demonstrated concentration-dependent vasorelaxation (Figure 8A). The vasorelaxation degree in the endothelium-intact group was slightly lower than that of the endothelium-denuded group, but the difference was not statistically significant (*p* > 0.05). This result indicated that PDG might relax blood vessels by directly acting on the vascular smooth muscle cells.

The aortic rings also showed concentration-dependent vasorelaxation with the increased concentration of PINL, as shown in Figure 8B. At the concentration of 50–100 μM, the vasorelaxation degrees in the endothelium-denuded group was significantly lower than that in the endothelium-intact group (*p* < 0.05). This result suggested that the vasorelaxant effects of PINL might be endothelium dependent. The vascular response to Ach was used to examine whether the endothelium was completely removed (Figures 8C,D).

As shown in Figure 8E, the aortic rings represented a concentration-dependent vasorelaxant effects in all groups. At a concentration of 50 μM, the vasorelaxant response in PINL group was significantly better than in the PDG group (*p* < 0.05). Compared with single PDG and PINL groups, the combination group (PDG: PINL = 1:1) showed significantly enhanced vasorelaxant effects (*p* < 0.05) at all concentrations (25, 50, and 100 μM).

## Discussion

In this study, the ADME properties of PDG and PINL, including the kinetic solubility, permeability, plasma protein binding rate, and metabolic stability in liver microsomes, were systematically investigated.

In drug discovery, solubility has a major impact on bioassays, formulation for *in vivo* dosing, and intestinal absorption, where it translates directly into their pharmacokinetics, pharmacodynamics, and toxicodynamics. Kinetic solubility assays are often performed in high throughput with short incubation times and high throughput analyses using plate readers in the early discovery phases, which is useful for rapid compound assessment, guiding optimization via structure modification, and diagnosing bioassays (Kerns et al., 2008). Solubility of less than 1 µM is regarded as highly insoluble, that between 1 and 100 µM is regarded as partially soluble, and that more than 100 µM is regarded as soluble. Therefore, based on this criterion, the kinetic solubilities in PBS (pH 7.4) of both PDG and PINL were greater than 100 μM, which indicated that they may be classified as soluble components. The kinetic solubility of PDG was slightly higher than that of PINL, indicating the glycosylation results in glycosides with increased solubility compared with the corresponding aglycone (Sun et al., 2020).

PAMPA is a method for predicting passive intestinal absorption and has been regarded as a helpful complement and alternative to Caco-2 assay in pharmaceutical research in the last decades (Piazzini et al., 2017). In the present study, the results of permeability investigations indicated that PINL had higher permeability than PDG. PINL had a higher *P*_*eff.*_ value in PAMPA, which indicated a strong transmembrane capacity. This result is consistent with traditional views that the components with larger molecular weight (PDG) are relatively weak in transmembrane capacity.

Serum albumin is a transport and deposition protein and serves as a carrier and storage for many endogenous and exogenous molecules, including fatty acids, amino acids, steroid hormones, metal ions, drugs, and many other molecules in plasma and affects the pharmacokinetic parameters of many medicinal molecules. The protein binding rate affects the distribution, excretion, metabolism, and interaction of some medicinal molecules with tissues, thereby influencing their bioavailability (Cao et al., 2019). Plasma protein binding rate represents the degree to which medications attach to proteins within the blood, which is a crucial parameter to determine the extent and duration of the therapeutic action of a drug; it is useful in the design of effective drug delivery schemes and estimation of safe dose (Wang et al., 2016). PDG and PINL represented moderated plasma protein binding properties, where PDG represented a high protein binding rate in either human plasma or rat plasma. Based on the results of a plasma protein binding study, the glycosylation of PINL decreased the protein binding rate by 20–40%. Similar to the results of the glycosylation of flavonoids, this decline in protein binding rate might attributed to steric hindrance that limits the capacity to enter the hydrophobic pockets in HSA (Cao et al., 2019). Given that the glycosylated lignanoid (PDG) has better water solubility than its aglycone (PINL), the crucial driving force for the binding of PDG to HSA might be the polarity of the molecule.

In addition, the results of pharmacokinetic and urinary excretion studies indicated that PINL might be eliminated less quickly than PDG from the rat plasma, and its cumulative urinary excretion was much lower than that of PDG. This result might be attributed to their difference in plasma protein binding and permeability property. In the pharmacokinetic study, the results also indicated that the bioavailability of PDG (1.51%) by oral administration is much lower than that of PINL (38.38%). Along with their difference found in the ADME properties, PINL showed higher permeability and protein binding rate than PDG, which might contribute to its relatively higher oral bioavailability. However, the further application of PDG might be focus on the enhancement on the solubility or permeability of PDG to promote its oral bioavailability, such as proper pharmaceutical modifications (nanoparticles or liposomes).

Moreover, a double peak was found in the concentration–time profile of PINL oral administration, which might be attributed to other mechanisms (Cong et al., 2016), enterohepatic circulation, or fractionated gastric emptying and separated “absorption windows.” The mechanism of this absorption should be further investigated to understand the PINL property. The considerable difference between their properties in urinary excretion might be related to the difference in their plasma protein binding activities, where PINL showed a higher plasma protein binding rate than PDG. The high plasma protein binding might have led to reduced metabolism and renal filtration, which resulted in a decrease in overall clearance. In addition, the difference in the permeability properties of these compounds is another possible reason. Better permeability related to the molecular structure (high lipophilic property) might lead to a low level of excretion.

Based on the results of vasorelaxant effects, all of the groups, including the single PDG group, single PINL group, and PDG + PINL groups, showed concentration-dependent vasorelaxant effects on PE-constricted blood vessels. The vasorelaxant effects of PINL were significantly better than those of PDG, whereas the vasorelaxant effect of their combination (PDG + PINL) was significantly better than that of the single component (*p* < 0.05). The advantage of the combination in vasorelaxant effects also provide strong evidence for the multi-component and multi-target effects of natural products, including Chinese medicinal plants. However, a study (Kwan et al., 2003) reported that the aqueous extracts of the herb represented endothelium-dependent vasorelaxant effects, and the *in vitro* vasorelaxant action of *E. ulmoides* Oliv. might serve as the pharmacological basis for its well-documented antihypertensive action. The present study found that PDG might relax blood vessels by directly acting on the vascular smooth muscle cells, whereas PINL might be endothelium dependent. Therefore, the comprehensive vasorelaxant effects of Eucommiae cortex might depend on the amount of the total lignans in the herb, and the proportion of the PDG and PINL within them.

However, the core importance of the present study is actually to compare the ADME properties and the pharmacokinetic performance of PDG and PINL, which might be related to the similarity and difference of their chemical structures. These results involves the properties of the lignans in the very early stage in the drug-like discovery. Much more efforts are still needed in the next stage, and there will be a long way to go before the lignan (PDG or PINL) can be regarded as an independent potential new drug.

## Conclusion

This work investigated the *in vitro* ADME properties of PDG and PINL using molecular modeling methods. The results of kinetic solubility in PBS (pH 7.4) of both PDG and PINL were greater than 100 μM, which indicated that they were soluble compounds. The permeability investigations *via* PAMPA indicated that PINL had a higher permeability than PDG. They both represent moderate plasma protein binding properties (binding rate ranged from 37.69 to 89.03%) and low metabolic rate either in HLM (*t*_*1/2*_ ranged from 1,004.8 to 1,509.5 min) or in RLM (*t*_*1/2*_ ranged from 149.7 to 1,035.2 min). Furthermore, the results of pharmacokinetic and urinary excretion studies indicated that PINL might be eliminated less quickly than PDG from rat plasma, and its cumulative urinary excretion was much lower than PDG, which might be due to the difference in their plasma protein binding and permeability properties. The results of vasorelaxant effects indicated that PDG might relax blood vessels by directly acting on the vascular smooth muscle cells, whereas the effects of PINL might be endothelium dependent. The study on the ADME properties of PINL and PDG, and their vasorelaxant effects on phenylephrine-induced model, might provide important information for their possibility as potential drugs originating from natural products and their pharmaceutical research.

## Data Availability

The original contributions presented in the study are included in the article/Supplementary Material, further inquiries can be directed to the corresponding authors.
