# Supplementary material for: Comparison of Pinoresinol and its Diglucoside on their ADME Properties and Vasorelaxant Effects on Phenylephrine-Induced Model
Source: Front Pharmacol. 2021 Aug 9;12:695530. doi: 10.3389/fphar.2021.695530 (PMC8381248; doi:10.3389/fphar.2021.695530)
Supplement: Supplementary file 1 [file DataSheet1.pdf]

Support Materials
(Supplement 1-5)

Supplement 1: LC-MS/MS conditions in "Kinetic solubility test"

(1) HPLC parameters for PDG

HPLC system	Shimadzu LC-20AD		
chromatographic column	Thermo Betasil, C18,2.1×50 mm, 5µm		
column temperature	25 °C		
flow rate	0.5 mL/min		
mobile phase	Phase A: water with 0.1 % formic acid<br>Phase B: ACN with 0.1 % formic acid		
guard column	C18, 4×3.0mm, 5µm		
gradient condition	Time (min)	A (%)	B (%)
	0.01	98	2
	0.3	98	2
	1.5	5	95
	1.9	5	95
	2	98	2
	2.5	98	2

(2) HPLC parameters for PINL

HPLC system	Shimadzu LC-20AD		
chromatographic column	Thermo Betasil, C18,2.1×50 mm, 5µm		
column temperature	25 °C		
flow rate	0.5 mL/min		
mobile phase	Phase A: water with 0.1 % formic acid<br>Phase B: ACN with 0.1 % formic acid		
guard column	C18, 4×3.0mm, 5µm		
gradient condition	Time (min)	A (%)	B (%)
	0.01	85	15
	0.1	85	15
	1.5	5	95
	1.9	5	95
	2	85	15
	2.5	85	15

(3) HPLC parameters for nicardipine

HPLC system	Shimadzu LC-20AD
chromatographic column	Thermo Betasil, C18,2.1×50 mm, 5µm
column temperature	25 °C
flow rate	0.5 mL/min

mobile phase	Phase A: water with 0.1 % formic acid<br>Phase B: ACN with 0.1 % formic acid		
guard column	C18, 4×3.0mm, 5μm		
gradient condition	Time (min)	A (%)	B (%)
	0.01	85	15
	0.1	85	15
	1.5	5	95
	1.9	5	95
	2	85	15
	2.5	85	15

(4) HPLC parameters for ketoconazole

HPLC system	Shimadzu LC-20AD		
chromatographic column	Thermo Betasil, C18,2.1×50 mm, 5μm		
column temperature	25 °C		
flow rate	0.5 mL/min		
mobile phase	Phase A: water with 0.1 % formic acid<br>Phase B: ACN with 0.1 % formic acid		
guard column	C18, 4×3.0mm, 5μm		
gradient condition	Time (min)	A (%)	B (%)
	0.01	85	15
	0.1	85	15
	1.5	5	95
	1.9	5	95
	2	85	15
	2.5	85	15

(5) MS parameters

Compound	Ionization mode	Parent ion/Daughter ion	DP	CE	CXP	CAD	CUR (N_2)	GS1 (N_2)	GS2 (N_2)	IS	TEM	EP
Tolbutamide (IS)-1	ESI, MRM (-)	269.1/105.9	-40	-43	-15	10	20	40	50	-4500	500	-10
PDG	ESI, MRM (-)	681.3/519.2	-118	-18	-15	10	20	40	50	-4500	500	-10
Tolbutamide (IS)-2	ESI, MRM (+)	271.1/155	42	26	13	10	20	40	50	5500	500	10
PINL	ESI, MRM (+)	359.3/341.2	95	5	13	10	20	40	50	5500	500	10
nicardipine	ESI, MRM (+)	480.1/315.2	95	31	13	10	20	40	50	5500	500	10
ketoconazole	ESI, MRM (+)	531.2/135.3	120	53	13	10	20	40	50	5500	500	10
Propranolol (IS)	ESI, MRM (+)	260.3/116.2	70	24	13	10	20	40	50	5500	500	10

Supplement 2: LC-MS/MS conditions in "PAMPA"

Compound:	PDG, PINL			
HPLC:	Shimadzu LC30AD			
Autosampler:	Shimadzu SIL30ACMP			
Mass Spectrometer:	Qtrap 5500			
LC Method				
Column:	Thermo Betasil, C18 2.1×50mm, 5um			
Mobile Phase:	A: 0.1% formic acid in water; B: 0.1% FA in ACN			
Total Time(min)	Flow Rate (μL/min)	A (%)	B (%)	
0.01	600	70	30	
0.10	600	70	30	
1.00	600	2	98	
2.10	600	2	98	
2.20	600	70	30	
2.60	600	70	30	
Mass Spectrometer Conditions				
Ionization Mode:	ESI			
Compound ID:	Q1	Q3	DP	CE
Tol(IS)	271.1	155	42	26
PDG	681.3	519.4	-120	-17
PINL	357.3	151	-95	-23

Supplement 3: LC-MS/MS conditions in "Plasma protein binding rate"

(1) HPLC parameters for PDG and tolbutamide

HPLC system	Agilent 1200		
chromatographic column	Thermo Betasil, C18,2.1×50 mm, 5μm		
column temperature	25 °C		
flow rate	0.5 mL/min		
mobile phase	Phase A: water with 0.1 % formic acid<br>Phase B: ACN with 0.1 % formic acid		
guard column	C18, 4×3.0mm, 5μm		
gradient condition	Time (min)	A (%)	B (%)
	0.01	90	10
	0.2	90	10
	2.3	5	95
	2.8	5	95
	2.9	90	10
	3.5	90	10

(2) HPLC parameters for PINL, midazolam and tolbutamide

HPLC system	Agilent 1200		
chromatographic column	Thermo Betasil, C18,2.1×50 mm, 5μm		
column temperature	25 °C		
flow rate	0.5 mL/min		
mobile phase	Phase A: water with 0.1 % formic acid<br>Phase B: ACN with 0.1 % formic acid		
guard column	C18, 4×3.0mm, 5μm		
gradient condition	Time (min)	A (%)	B (%)
	0.01	75	25
	0.10	75	25
	1.80	10	90
	2.30	10	90
	2.40	75	25
	3.00	75	25

(3) HPLC parameters for diclofenac, propranolol, midazolam and tolbutamide

HPLC system	Agilent 1200
chromatographic column	Thermo Betasil, C18,2.1×50 mm, 5μm
column temperature	25 °C
flow rate	0.5 mL/min
mobile phase	Phase A: water with 0.1 % formic acid<br>Phase B: ACN with 0.1 % formic acid
guard column	C18, 4×3.0mm, 5μm

gradient condition	Time (min)	A (%)	B (%)
	0.01	70	30
	0.1	70	30
	2.8	5	95
	3.5	5	95
	3.6	70	30
	5	70	30

(4) MS parameters

Compound	Ionization mode	Parent ion/Daughter ion	DP	CE	CXP	CAD	CUR (N_2)	GS1 (N_2)	GS2 (N_2)	IS	TEM	EP
Tolbutamide (IS)-1	ESI, MRM (-)	269.1/105.9	-40	-43	-16	Medium	20	18	0	-4500	0	-10
PDG	ESI, MRM (-)	681.3/519.2	-200	-19	-16	Medium	20	18	0	-4500	0	-10
Tolbutamide (IS)-2	ESI, MRM (+)	271.1/155	42	26	15	4	10	12	0	4500	0	10
Midazolam (IS)-1	ESI, MRM (+)	326/291.4	115	34	15	4	10	12	0	4500	0	10
PINL	ESI, MRM(+)	359.3/341.2	95	5	15	4	10	12	0	4500	0	10
Tolbutamide (IS)-3	ESI, MRM (+)	271.1/155	42	26	16	Medium	20	18	0	5500	0	10
Midazolam (IS)-2	ESI, MRM (+)	326/291.4	115	34	16	Medium	20	18	0	5500	0	10
diclofenac	ESI, MRM (+)	296/215.2	120	30	16	Medium	20	18	0	5500	0	10
propranolol	ESI, MRM (+)	260.3/116.2	70	24	16	Medium	20	18	0	5500	0	10

Sample volume:

PDG, PINL, diclofenac: 20 µL;

propranolol: 8 µL.

Supplement 4: LC-MS/MS conditions in “Metabolic stability of liver microsomes”

(1) HPLC parameters for PINL, dextromethorphan, omeprazole, and tolbutamide, and midazolam

HPLC system	Shimadzu LC-20AD		
chromatographic column	Thermo Betasil, C18,2.1×50 mm, 5μm		
column temperature	25 °C		
flow rate	0.5 mL/min		
mobile phase	Phase A: water with 0.1 % formic acid;<br>Phase B: ACN with 0.1 % formic acid		
gradient condition	Time (min)	A (%)	B (%)
	0.01	75	25
	0.10	75	25
	1.80	10	90
	2.30	10	90
	2.40	75	25
	3.00	75	25

(2) HPLC parameters for PDG and tolbutamide

HPLC system	Shimadzu LC-20AD		
chromatographic column	Thermo Betasil, C18,2.1×50 mm, 5μm		
column temperature	25 °C		
flow rate	0.5 mL/min		
mobile phase	Phase A: water with 0.1 % formic acid;<br>Phase B: ACN with 0.1 % formic acid		
gradient condition	Time (min)	A (%)	B (%)
	0.01	95	5
	0.10	95	5
	1.10	10	90
	1.70	10	90
	1.80	95	5
	2.00	95	5

(3) MS parameters

Compound	Ionization mode	Parent ion/Daughter ion	DP	CE	CXP	CAD	CUR (N_2)	GS1 (N_2)	GS2 (N_2)	IS	TEM	EP
Tolbutamide (IS)-1	ESI, MRM(+)	271.1/155	42	26	15	4	10	12	0	4500	0	10
Midazolam (IS)	ESI, MRM(+)	326/291.4	115	34	15	4	10	12	0	4500	0	10
Dextromethorphan	ESI, MRM(+)	271.9/147.1	60	42	15	4	10	12	0	4500	0	10
Omeprazole	ESI,	346.1/197.9	59	17	15	4	10	12	0	4500	0	10

	MRM(+)											
PINL	ESI, MRM(+)	359.3/341.2	95	5	15	4	10	12	0	4500	0	10
Tolbutamide (IS)-2	ESI, MRM(-)	269.1/105.9	-40	-43	-15	6	10	10	0	-4500	0	-10
PDG-1	ESI, MRM(-)	681.3/519.3	-118	-18	-15	6	10	10	0	-4500	0	-10
PDG-2	ESI, MRM(-)	681.3/356.8	-118	-34	-15	6	10	10	0	-4500	0	-10

Supplement 5: LC-MS/MS conditions in "Pharmacokinetic and excretion studies"

(1) HPLC parameters for PDG for plasma and urine (IV)

HPLC system	Agilent 1200		
chromatographic column	Thermo Betasil, C18,2.1×50 mm, 5μm		
column temperature	25 °C		
flow rate	0.5 mL/min		
mobile phase	Phase A: water with 0.1 % formic acid<br>Phase B: ACN with 0.1 % formic acid		
gradient condition	Time (min)	A (%)	B (%)
	0.01	80	20
	1.5	10	90
	2.2	10	90
	2.3	80	20
	3.0	80	20

(2) HPLC parameters for PDG and PINL for plasma (IV)

HPLC system	Agilent 1200		
chromatographic column	Thermo Betasil, C18,2.1×50 mm, 5μm		
column temperature	25 °C		
flow rate	0.6 mL/min		
mobile phase	Phase A: water with 0.1 % formic acid<br>Phase B: ACN with 0.1 % formic acid		
gradient condition	Time (min)	A (%)	B (%)
	0.01	70	30
	1.5	2	98
	2.2	2	98
	2.6	70	30
	3.0	70	30

(3) HPLC parameters for PINL for urine (IV, PO)

HPLC system	Agilent 1200		
chromatographic column	Thermo Betasil, C18,2.1*50 mm, 5μm		
column temperature	25 °C		
flow rate	0.4 mL/min		
mobile phase	Phase A: water with 0.1 % Formic acid<br>Phase B: ACN with 0.1 % Formic acid		
gradient condition	Time (min)	A (%)	B (%)
	0.01	70	30
	0.5	50	50
	2.5	25	75
	2.8	5	95

	3.2	5	95
	3.3	70	30
	4.5	70	30

(4) MS parameters

Mass Detector: AB Qtrap5500

CTC: HTC PAL CTC Analytics

Compound	Ionization mode	Parent ion/Daughter ion	DP	CE	CXP	CAD	CUR (N_2)	GS1 (N_2)	GS2 (N_2)	IS	TEM	EP
PDG-1 (PO)	ESI, MRM(-)	681.3/519.3	-50	-18	-12	Medium	20	50	45	-4500	500	-10
PDG-2 (PO)	ESI, MRM(-)	681.3/357.3	-50	-30	-12	Medium	20	50	45	-4500	500	-10
PINL (PO)	ESI, MRM(-)	357.3/150.9	-80	-26	-12	Medium	20	50	45	-4500	500	-10
Tolbutamide (IS)-1	ESI, MRM(-)	269.1/105.9	-40	-43	-12	Medium	20	50	45	-4500	500	-10
diclofenac sodium (IS)-1	ESI, MRM(-)	294.0/250.0	-50	-14	-12	Medium	20	50	45	-4500	500	-10
PDG-1 (IV)	ESI, MRM (-)	681.3/519.3	-50	-18	-12	Medium	20	60	55	-4500	500	-10
PDG-2 (IV)	ESI, MRM (-)	681.3/357.3	-50	-30	-12	Medium	20	60	55	-4500	500	-10
PINL (IV)	ESI, MRM (-)	357.3/150.9	-80	-26	-12	Medium	20	60	55	-4500	500	-10
Tolbutamide (IS)-2	ESI, MRM(-)	269.1/105.9	-40	-43	-12	Medium	20	60	55	-4500	500	-10
diclofenac sodium (IS)-2	ESI, MRM(-)	294.0/250.0	-50	-14	-12	Medium	20	60	55	-4500	500	-10
